# Graphitic Carbon Nitride Confers Bacterial Tolerance to Antibiotics in Wastewater Relating to ATP Depletion

**DOI:** 10.3390/molecules29235780

**Published:** 2024-12-06

**Authors:** Shuo Liu, Lin Teng, Jiantao Ping

**Affiliations:** 1 School of Energy and Chemical Engineering, Tianjin Renai College, Tianjin 301636, China; 2 Shandong Analysis and Test Center, Qilu University of Technology (Shandong Academy of Sciences), Jinan 250014, China

**Keywords:** graphitic carbon nitride, antibiotic tolerance, ATP depletion, photocatalyst, wastewater

## Abstract

Graphitic carbon nitride (C_3_N_4_) is a kind of visible light-responsive photocatalyst that has been of great interest in wastewater treatment. However, its environmental impact and biological effect remains to be elucidated. This study investigated the effect of C_3_N_4_ nanosheets on bacterial abundance and antibiotic tolerance in wastewater. Interestingly, as compared to the wastewater containing the antibiotic ofloxacin alone, the wastewater containing both ofloxacin and C_3_N_4_ had much higher numbers of total living bacteria, but lower levels of the ofloxacin-resistant bacteria and the ofloxacin-resistant gene *qnrS*. The model bacterium *Staphylococcus aureus* was then used to explore the mechanism of C_3_N_4_-induced antibiotic tolerance. The nanosheets neither adsorbed the antibiotic nor promoted drug efflux, uncovering that drug adsorption and efflux were not involved in antibiotic tolerance. Further investigations revealed that the nanosheets, like arsenate and menadione, drastically reduced ATP levels and induced the production of reactive oxygen species for enhanced antibiotic tolerance. This study revealed an antibiotic-tolerating mechanism associated with C_3_N_4_-induced ATP depletion, and shed a light on the effect of photocatalysts on microbial ecology during their application in wastewater treatment.

## 1. Introduction

Photocatalysis technology, which converts renewable light energy into useful chemical and electric energy, is a promising solution to deal with wastewater contamination [1,2]. With the advance of nanotechnology, a variety of photocatalysts have been developed and utilized in the removal of organic pollutants and disinfection of pathogenic microbes [3,4,5]. Among them, graphitic carbon nitride (C_3_N_4_) is receiving significant attention as a metal-free photocatalyst, with the advantage of its unique physical and chemical properties, such as its visible-light-driven bandgap, tunable electron band structure, good biocompatibility and easy preparation [6,7]. Through the modification of microstructures and surfaces, the photocatalytic activity of C_3_N_4_ could be strongly improved, further expanding its potential applications in the fields of energy conversion and storage, biosensing, biomedicine, and especially environmental engineering [8,9,10].

The Antibiotic tolerance of natural bacteria is becoming a great concern in environmental science [11,12]. Owing to the severe release of antibiotics from hospitals, antibiotic factories, and livestock breeding places, water-cycling systems are suffering from severe antibiotic pollution. Consequently, more and more bacteria in wastewater are evolving and exhibit the capacity to tolerate antibiotics in wastewater, wastewater treatment plant effluents, and even natural water system [13]. A well-known mechanism contributing to antibiotic tolerance is the prevalent existence of antibiotic-resistant genes (ARGs) in natural bacterial cells, such as the quinolone-resistant gene *qnrS*, the multidrug efflux transporter-encoding genes *mexB* and *mexD*, and the β-lactam-resistant genes *ampC* and *bla_TEM_* [14]. These genes endow bacterial cells with resistance to different kinds of antibiotics by generating antibiotic-inactivating enzymes, drug efflux transporters, antibiotic-insensitive targets, etc. [15]. However, to this day, little is known about the ARG-independent mechanisms by which the natural bacterial cells tolerate antibiotics.

With the increasing discharge of nanomaterials in wastewater treatment, their environmental impacts are of great concern [16,17]. In terms of their effect on microbial ecology in water systems, a series of nanomaterials, such as nano-metal oxides, cellulose nanocomposites, silver nanoparticles, and graphene oxides, have been proven to inactivate microbial cells and to remove ARGs [18,19]. For example, nano-metal oxides disinfect drug-resistant bacteria and attenuate drug tolerance by direct attachment to bacterial cells and the dissolution of metal ions [20]. As described above, C_3_N_4_ is becoming a research hotspot in environmental engineering. However, its environment impacts, especially their effect on the crucial microbial population in wastewater ecosystems, still urgently need to be elucidated [21].

**Figure 1:**
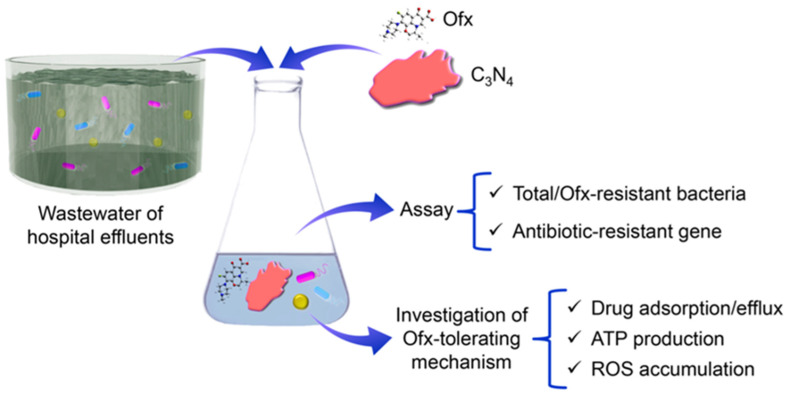
Illustration of the procedures for the investigation of the effect of C_3_N_4_ nanosheets on bacterial tolerance to antibiotics in wastewater. Ofx, ofloxacin.

In this study, we aimed to clarify the biological effect of C_3_N_4_ on microbial ecology in wastewater. The effects of the prepared nanosheets on the abundance of total and antibiotic-resistant bacteria, together with ARG levels, were investigated by using the wastewater of hospital effluents with the addition of the model antibiotic, ofloxacin (Ofx). The model bacterium, *Staphylococcus aureus*, was then used to explore the antibiotic-tolerating mechanism. A series of assays for antibiotic adsorption, antibiotic efflux, ATP production and reactive oxygen species (ROS) accumulation were performed to investigate the possible contribution of antibiotic transport and ATP production on the enhancement of antibiotic tolerance (Figure 1). This study sheds a light on biological effect of photocatalysts during environmental engineering.

## 2. Results and Discussion

### 2.1. Characterization

**Figure 2:**
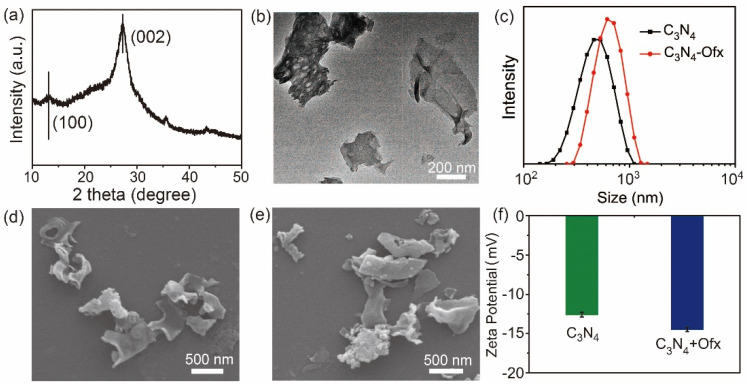
Characterization of C_3_N_4_ and C_3_N_4_+Ofx. (**a**) XRD pattern of C_3_N_4_. (**b**) TEM image of C_3_N_4_. (**c**) DLS analysis of C_3_N_4_ and C_3_N_4_+Ofx. (**d**) SEM image of C_3_N_4_. (**e**) SEM image of C_3_N_4_+Ofx. (**f**) Zeta potential of C_3_N_4_ and C_3_N_4_+Ofx.

The C_3_N_4_ nanosheets were prepared by using a previously reported method [22], and were characterized by XRD, TEM, SEM, DLS and Zeta potential analysis. All data supported the successful preparation of C_3_N_4_ nanosheets. The crystal structures of C_3_N_4_ were determined by XRD (Figure 2a), with two peaks at 13.1° and 27.4° corresponding to the (100) and (002) planes of C_3_N_4_ (JCPDS 87-1526) [23]. As shown by TEM and SEM images, the C_3_N_4_ nanosheets showed a sheet-like morphology with a lateral size of several hundred nanometers (Figure 2b,d), which was consistent with the hydration diameter obtained by DLS (Figure 2c).

As a typical kind of two-dimensional nanomaterial, we wonder if C_3_N_4_ could interact with the model antibiotic, Ofx, and further affect the antibacterial efficiency of the drug [24]. SEM, DLS and Zeta potential were also used to characterize the mixture of C_3_N_4_ and Ofx (C_3_N_4_+Ofx). As shown in Figure 2c, when mixing C_3_N_4_ and Ofx, the size of C_3_N_4_+Ofx ranged from 200 to 1200 nm, while the size range of C_3_N_4_ was 150–1100 nm, indicating that the introduction of Ofx did not significantly change the size of C_3_N_4_. Consistently, C_3_N_4_+Ofx also showed a sheet-like morphology with a lateral size of several hundred nanometers based on the SEM images (Figure 2e), similarly to C_3_N_4_ (Figure 2d). Moreover, the zeta potential of C_3_N_4_+Ofx was a little lower than that of C_3_N_4_, which is mainly attributed to the negative property of Ofx (Figure 2f).

In short, the above characterization results showed that the introduction Ofx did not significantly change the morphology, size or charge of C_3_N_4_.

### 2.2. Effect of C_3_N_4_ on Abundance of Bacteria in Antibiotic-Containing Wastewater

**Figure 3:**
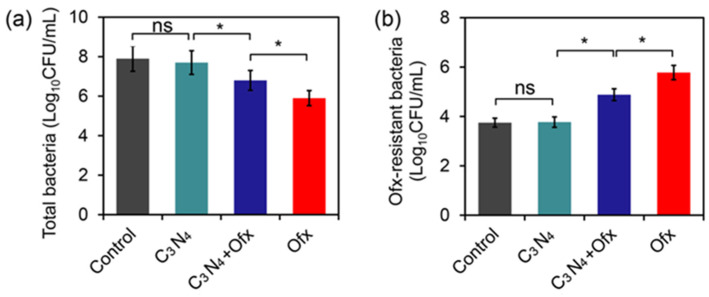
Effect of C_3_N_4_ and Ofx on bacterial abundance in wastewater. (**a**) Number of total living bacteria in the wastewater after 2 days of treatment with C_3_N_4_ (80 ppm), C_3_N_4_ (80 ppm) plus Ofx (16 ppm) or Ofx (16 ppm). (**b**) Number of Ofx-resistant bacteria in the wastewater. The asterisks (*) indicate significant differences between the groups, while “ns” indicates no significant difference (*p* < 0.05).

Since C_3_N_4_-based nanomaterials are frequently used as photocatalysts in research on antibiotic-containing wastewater treatment, we firstly investigated the impact of the obtained C_3_N_4_ nanosheets on the abundance of total bacteria and antibiotic-resistant bacteria in wastewater containing the model antibiotic Ofx. As shown in Figure 3, C_3_N_4_ alone did not reduce numbers of total and Ofx-resistant bacteria, indicating no obvious toxicity of the nanosheets to the natural bacteria in wastewater. In contrast, Ofx alone strongly reduced the number of total bacteria from 107.9 CFU/mL to 105.9 CFU/mL (Figure 3a), which was attributed to the antibacterial activity of Ofx. Interestingly, C_3_N_4_ plus Ofx only moderately reduced total bacterial numbers to 106.8 CFU/mL (Figure 3a). This indicated that the nanosheets remarkably increased the Ofx-tolerating capacity of the bacteria in wastewater.

To investigate whether the enhanced tolerance of bacteria was related to an increase in Ofx-resistant bacteria, the abundance of the resistant bacteria was evaluated. While the control and C_3_N_4_ group had very low levels of Ofx-resistant bacteria (103.7 CFU/mL and 103.8 CFU/mL), the C_3_N_4_+Ofx and Ofx groups displayed obvious, comparably high levels of the resistant bacteria (104.9 CFU/mL and 105.8 CFU/mL, Figure 3b). However, although the C_3_N_4_+Ofx group had much higher levels of total bacteria than the Ofx group, it displayed lower Ofx-resistant bacteria, indicating that the enhanced bacterial tolerance to Ofx by C_3_N_4_ was not attributed to an increase in Ofx-resistant bacteria.

### 2.3. Effect of C_3_N_4_ on ARG Levels in Antibiotic-Containing Wastewater

**Figure 4:**
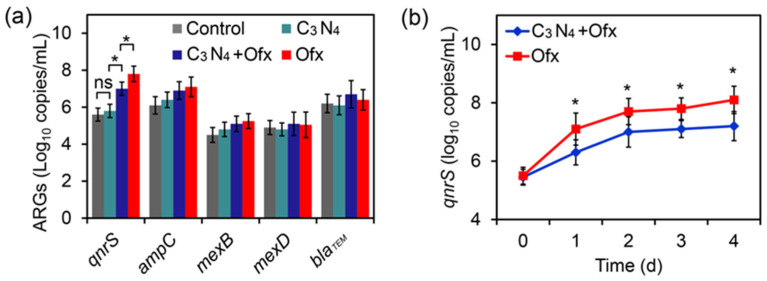
Effect of C_3_N_4_ and Ofx on the levels of antibiotic-resistant genes (ARGs). (**a**) Levels of the representative ARGs after 2 days of treatment. (**b**) Levels of the *qnsR* gene at the indicated treating time points in the different groups. The asterisks (*) indicate significant differences between the groups, while “ns” indicates no significant difference (*p* < 0.05).

To further confirm that Ofx-resistant bacteria did not contribute to the C_3_N_4_-induced enhancement of bacterial Ofx tolerance in the wastewater, a series of ARGs were detected by the real-time PCR method. After 2 days of treatment, both the C_3_N_4_+Ofx and Ofx groups had a drastic increase in the levels of *qnrS*, a well-known ARG involved in Ofx resistance [14], while the C_3_N_4_ groups maintained low levels of this gene as the control group (Figure 4a). Meanwhile, the four groups displayed similar levels of *ampC*, *mexB*, *mexD*, and *bla_TEM_* (Figure 4a), which are involved in resistance to other antibiotics, indicating that Ofx only led to an increase in ARGs involved in Ofx resistance.

The time-dependent change in *qnrS* levels was further evaluated in the C_3_N_4_+Ofx and Ofx groups. With the increase in treating time, the *qnrS* levels exhibited an ascending trend in both groups. In the C_3_N_4_+Ofx group, the levels increased from 105.5 copies/mL to 107.2 copies/mL, while those in the Ofx group increased to 108.1 copies/mL (Figure 4b), indicating that the C_3_N_4_+Ofx group had much lower *qnrS* levels than the Ofx group. Together, these results revealed the inhibitory effect of the C_3_N_4_ nanosheets on the increase in Ofx-resistant genes under Ofx treatment, further confirming that the C_3_N_4_-induced enhancement of bacterial Ofx tolerance in the wastewater was not attributed to the presence of Ofx-resistant bacteria.

### 2.4. Effect of C_3_N_4_ on Antibacterial Efficiency of Ofx

**Figure 5:**
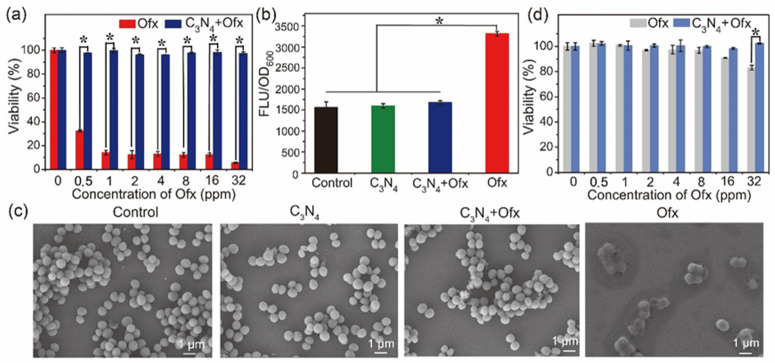
Effects of C_3_N_4_ on the antibacterial efficiency of Ofx against normal SA and ORSA. (**a**) Viability of normal SA after 24 h of incubation with different concentrations of Ofx and C_3_N_4_+Ofx. (**b**) Membrane depolarization induced by the agents. The higher fluorescence intensity indicates a higher degree of depolarization. (**c**) SEM images of SA with different treatments. (**d**) Viability of ORSA after 24 h of incubation with different concentrations of Ofx and C_3_N_4_+Ofx. The asterisks (*) indicate significant differences between the groups (*p* < 0.05).

To further investigate the mechanism of C_3_N_4_-induced Ofx tolerance, the common bacterium *S. aureus* was used for further biochemical analysis. As shown in Figure 5a, Ofx strongly inhibited the growth of the Ofx-sensitive *S. aureus* strain SA, with less than 20% of SA surviving in the presence of 1 ppm Ofx. However, in the presence of 80 ppm C_3_N_4_, the growth of SA was unaffected by Ofx even at a higher concentration (32 ppm). The results were consistent with the results of cell membrane potential analysis with different treatments. As shown in Figure 5b, the membrane potential of SA treated with C_3_N_4_ or C_3_N_4_+Ofx was the same as that of the control group, while the degree of membrane potential depolarization of SA treated with Ofx was significantly higher than that of the other groups, demonstrating that only Ofx depolarized the membrane of SA [25]. Furthermore, the morphological change in SA after different treatments was observed by SEM. As shown in Figure 5c, most of the SA cells maintained a normal shape like the control cells in both C_3_N_4_- and C_3_N_4_+Ofx-treated groups, indicating that C_3_N_4_ or C_3_N_4_+Ofx failed to kill the bacteria, and the C_3_N_4_ nanosheets were apparently attached to the surface of the bacteria. In contrast, the bacterial cells treated by Ofx were drastically wrinkled with an abnormal morphology, further confirming the attenuation of the antibacterial efficiency of Ofx by C_3_N_4_.

ORSA, an Ofx-resistant *S. aureus* strain, was also used for drug tolerance assays. Obviously, Ofx at 0~16 ppm had no obvious impact on the growth of ORSA, while Ofx at 32 ppm decreased the viability of the bacterial cells by 20% (Figure 5d). Consistent with the results of the normal SA, the inhibition efficiency of Ofx at 32 ppm was almost diminished by the addition of C_3_N_4_ (Figure 5d). Moreover, pure C_3_N_4_ had no impact on the growth of SA or ORSA (Figure S1). These results revealed that C_3_N_4_ improved the Ofx tolerance of both SA and ORSA.

### 2.5. Effect of C_3_N_4_ on Antibacterial Efficiency of Ofx During Bacterium–Macrophage Interaction

To evaluate the effect of C_3_N_4_ on the antibacterial efficiency of Ofx during pathogen infection, a bacterium–macrophage interaction model was firstly used by using SA and RAW 264.7 macrophages. Firstly, the cytotoxicity of C_3_N_4_ and Ofx was assessed by CCK-8 assays. As shown in Figure S2, both C_3_N_4_ and Ofx had no obvious toxicity to the RAW 264.7 macrophages in the experimental concentration (i.e., up to 150 ppm for C_3_N_4_ and 32 ppm for Ofx).

**Figure 6:**
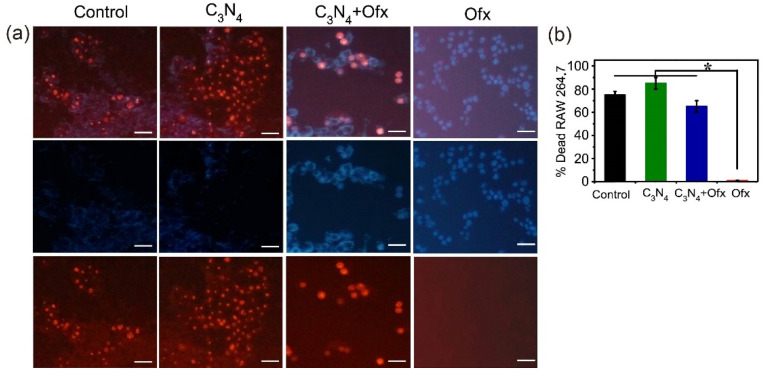
Impairment of antibiotic efficiency against SA during SA–macrophage interaction. (**a**) Confocal images of the RAW 264.7 cells infected by SA together with different treatments after 4 h. Scale bar = 50 μm. (**b**) The death rate of RAW 264.7 cells after 4 h of co-incubation. The asterisks (*) indicate significant differences (*p* < 0.05).

SA and RAW 264.7 macrophages were then co-incubated with different treatments for 4 h for confocal microscopy. As shown in Figure 6a, abundant macrophages died after co-incubation with SA without any treatment, indicating that SA could severely damage the RAW 264.7 cells. On the contrary, most of the RAW 264.7 cells survived in the presence of Ofx, suggesting that SA was strongly inhibited by Ofx. However, most of the RAW 264.7 cells also died in the presence of C_3_N_4_, even with the addition of Ofx. Statistical analysis further showed that the presence of C_3_N_4_ significantly attenuated the macrophage-protecting capacity of Ofx (Figure 6b).

### 2.6. Mechanisms of C_3_N_4_-Induced Impairment of Antibiotic Efficiency

**Figure 7:**
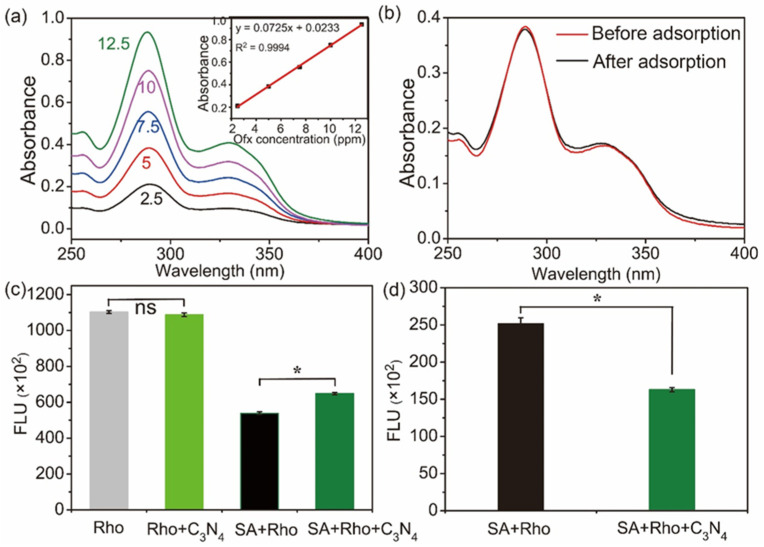
Ofx adsorption by C_3_N_4_ (**a**,**b**), and the effect of C_3_N_4_ on Ofx uptake (**c**) and efflux (**d**) by SA cells. (**a**) UV-Vis spectra of Ofx solutions with increased concentrations (insert: calibration curve obtained from the absorbance at 289 nm against the corresponding concentration of Ofx; the curve was fitted with a linear function.). (**b**) UV-Vis spectra of the Ofx solutions before and after C_3_N_4_ adsorption. (**c**) Rhodamine 6G (Rho) uptake by SA cells in the absence (SA+Rho) and presence (SA+Rho+C_3_N_4_) of C_3_N_4_. The groups of Rho and Rho+C_3_N_4_ represent the SA-free treatments. (**d**) Rho efflux from the SA cells in the absence and presence of C_3_N_4_. The asterisks (*) indicate significant differences, and “ns” indicates no significance between the groups (*p* < 0.05).

Antibiotic adsorption by two-dimensional nanomaterials frequently impairs the antibacterial efficiency of antibiotics [26]. As a typical kind of two-dimensional nanomaterial, the antibiotic adsorption capacity of C_3_N_4_ was first evaluated. To quantify the amount of Ofx adsorbed on C_3_N_4_, UV absorption of Ofx was used. As shown in Figure 7a, the absorption spectra of Ofx at a series of concentrations were obtained, and then a calibration curve was established by absorption at 289 nm, which could be linearly fitted (Figure 7a, insert). After incubation of C_3_N_4_ and Ofx for 24 h, the amount of un-adsorbed Ofx in the supernatant was calculated using its absorbance at 289 nm and the fitted formula from Figure 7a,b. As shown in Figure 7b, it is obvious that quite low levels of Ofx were adsorbed on C_3_N_4_ at room temperature and a neutral pH. We also evaluated the Ofx adsorption ability at 37 °C or other pHs (6 and 8), and similar results were obtained. Therefore, the adsorption of Ofx was not the reason for the weakened antibacterial effect of Ofx.

The adsorption ability of chloramphenicol, another typical antibiotic, was also evaluated. Similarly, the results showed that chloramphenicol was hardly adsorbed on C_3_N_4_ as well (Figure S3). The above results indicate that the C_3_N_4_-induced impairment of antibiotic efficiency was not caused by antibiotic adsorption by C_3_N_4_.

Uptake inhibition and drug efflux are two common antibiotic tolerance mechanisms of bacteria [27]. To test whether C_3_N_4_ affects Ofx uptake and efflux, rhodamine 6G (Rho), a fluorescent dye that is internalized and expelled by the same systems as those of Ofx in bacterial cells [28,29], was used for uptake and efflux assays. As shown in Figure 7c, while free C_3_N_4_ did not influence the fluorescence intensity of Rho, the uptake of Rho into the bacterial cells was not impaired, and was even slightly enhanced by C_3_N_4_.

Rho efflux was further evaluated by the incubation of the Rho-stained cells (SA with or without C_3_N_4_) in PBS containing 4 mM glucose for another 1 h. As shown in Figure 7d, the efflux of Rho was much higher in the absence of C_3_N_4_. Combined with the results of Figure 7c,d, C_3_N_4_ neither inhibited drug uptake into the bacterial cells nor promoted drug efflux from the cells. Therefore, drug uptake and efflux were not involved in the enhanced Ofx tolerance of SA induced by the C_3_N_4_ nanosheets.

**Figure 8:**
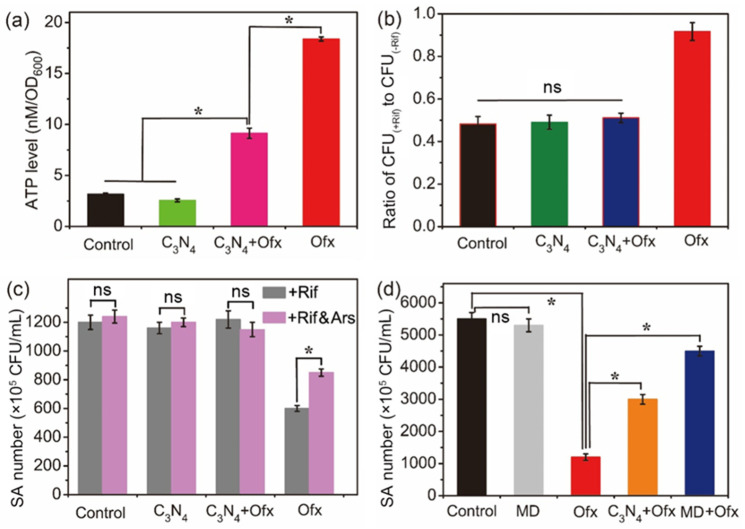
The effect of C_3_N_4_, Ars and MD on intracellular ATP levels (**a**) and SA viability (**b**–**d**). (**a**) ATP levels of SA with different treatments. (**b**) The ratio of CFU in the presence of Rif (CFU_(+Rif)_) to CFU in the absence of Rif (CFU_(-Rif)_) with different treatments. (**c**) The effect of Ars on living SA numbers. (**d**) The effect of MD on living SA numbers. The asterisks (*) indicate significant differenced, and “ns” indicates no significance between the groups (*p* < 0.05).

ATP is the direct energy source in living bacterial cells and has a significant effect on respiration, metabolism, and enzymatic reactions [30]. The ATP levels of SA with different treatments were then evaluated. As shown in Figure 8a, the ATP level of SA without any treatment (Control) reached 3.16 nM per OD_600_, a little higher than that of the C_3_N_4_-treated group (2.55 nM per OD_600_). Interestingly, the ATP level of SA treated with Ofx was the highest, whereas the level was strongly reduced by C_3_N_4_ in the Ofx-treated cells. This result suggested that the Ofx tolerance of SA in the C_3_N_4_+Ofx group was associated with reduced ATP levels, which is consistent with the previous reports [31,32]. Since electron transport on the bacterial plasma membrane is critical for ATP production [33], and C_3_N_4_ is a well-known photocatalyst with a strong capacity for electron transport [34], the ATP depletion caused by C_3_N_4_ was most likely attributed to electron shuttling from the plasma membrane to the nanosheets.

Rifampicin (Rif) is an antibacterial agent due to the inhibition of RNA transcription [35]. To test whether or not C_3_N_4_-induced Ofx tolerance is related to an alteration in transcriptional profiling, SA was pre-incubated with Rif and then treated with PBS, C_3_N_4_, C_3_N_4_+Ofx and Ofx for CFU assays. As shown in Figure 8b, the pre-incubation of SA with Rif led to a comparable decrease in the ratio of CFU among the control, C_3_N_4_ and C_3_N_4_+Ofx groups, indicating that C_3_N_4_-induced Ofx tolerance was not associated with an alteration in transcription profiling [36].

ATP levels could be depleted by arsenate (Ars) through the rapid formation of hydrolysable ADP-As [37]. To evaluate whether ATP depletion was related to C_3_N_4_-induced Ofx tolerance, Ars was either added or not added to PBS (control), C_3_N_4_, C_3_N_4_+Ofx and Ofx after the pre-incubation of Rif for CFU assays. For the control, C_3_N_4_ and C_3_N_4_+Ofx groups, the addition of Ars had no obvious impact on the viability of SA (Figure 8c). However, like C_3_N_4_, Ars remarkably improved the number of living SA under Ofx treatment (Figure 8c). These results indicated that ATP depletion was associated with the C_3_N_4_-induced Ofx tolerance of SA.

Menadione (MD) is a redox-cycling agent that induces oxidative stress and consequent Ofx tolerance [38]. To compare the effect of MD with that of C_3_N_4_, the SA cells were treated with MD, Ofx, C_3_N_4_+Ofx and MD+Ofx for CFU assays. As shown in Figure 8d, while MD (10 ppm) had no effect on the growth of SA, it significantly improved the survival rate of SA suffering from Ofx attack, which was similar to the effect of C_3_N_4_. Based on the above results, it is reasonable to conclude that C_3_N_4_ strongly decreased the ATP levels of SA for the induction of Ofx tolerance.

**Figure 9:**
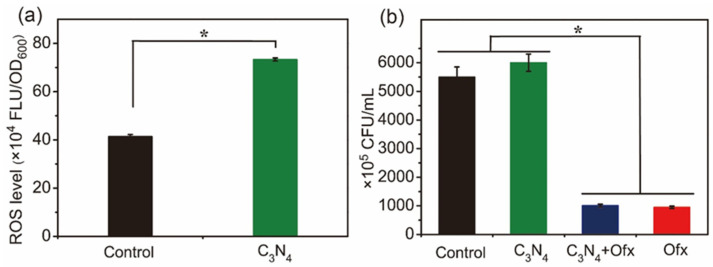
The effect of C_3_N_4_ and the ROS scavenger AAS on the ROS levels (**a**) and viability of SA (**b**). (**a**) ROS levels of SA in the absence and presence of C_3_N_4_. (**b**) CFUs of the SA cells under treatment with the agents in the presence of AAS. The asterisks (*) indicate significant differences between the groups (*p* < 0.05).

Reactive oxygen species (ROS) are the side products of respiration during the inhibition of electron transport and the ATP depletion of bacterial cells [38]. To test whether ATP depletion by C_3_N_4_ is also followed by ROS accumulation, the ROS levels of SA in the absence and presence of C_3_N_4_ were evaluated. As shown in Figure 9a, the ROS level of SA in the presence of C_3_N_4_ was much higher than that of the control group, suggesting that the lowered ATP levels were accompanied by ROS generation.

Sodium ascorbate (AAS), a common antioxidant used for ROS scavenging [39], was further used for co-incubation with C_3_N_4_. As shown in Figure 9b, the CFU of C_3_N_4_+Ofx was the same as that of the Ofx group in the presence of AAS, while the growth in the control and C_3_N_4_ group was not affected by AAS, indicating that ROS scavenging could not attenuate the antibacterial efficiency of Ofx. These results suggest that ROS accumulation was just a consequence of ATP depletion and Ofx tolerance, rather than a reason for the Ofx tolerance induced by the C_3_N_4_ nanosheets.

**Figure 10:**
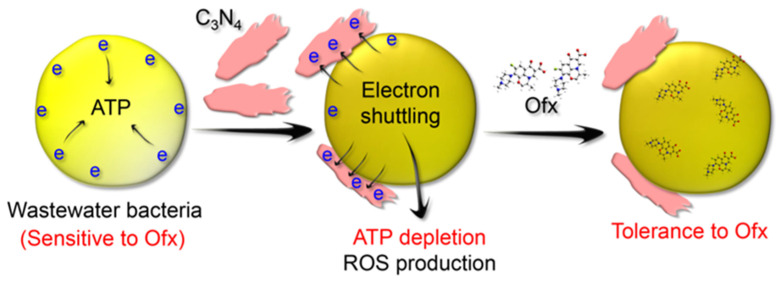
Illustration of the mechanism by which the C_3_N_4_ nanosheets enhance bacterial tolerance to the antibiotic Ofx. The blue letter “e” indicates electrons.

Based on the above results, we propose that the C_3_N_4_ nanosheets strongly enhance the tolerance of Ofx-sensitive bacterial cells to Ofx in wastewater in an ATP-related mechanism (Figure 10). Owing to their strong electron transporting capacity, the C_3_N_4_ nanosheets induced severe electron shuttling from the bacterial plasma membrane to the nanosheets, leading to ATP depletion of the bacterial cells and ROS production. Therefore, ATP-depleted bacterial cells, even though they do not possess ARGs and are inherently sensitive to the antibiotics, become highly tolerant to the antibiotics and survive in antibiotic-containing wastewater.

## 3. Materials and Methods

### 3.1. Chemicals and Reagents

Melamine, ofloxacin, rhodamine 6G, chloramphenicol, glucose, rifampicin, arsenate, menadione, sodium ascorbate, and Hoechst 33342 were purchased from Sigma, St. Louis, MO, USA. The Ofx-sensitive *S. aureus* strain NKS12 (SA) and Ofx-resistant *S. aureus* strain NKS13 (ORSA) were isolated from the clinic and stored in the Laboratory of Modern Mycology, Nankai University.

### 3.2. Preparation of C_3_N_4_ Nanosheets

Bulk C_3_N_4_ was synthesized by the thermal polycondensation of melamine [22]. Briefly, 4 g of melamine was maintained at 550 °C in a muffle furnace for 4 h. After cooling down to ambient temperature, the yellow bulk C_3_N_4_ was obtained.

C_3_N_4_ nanosheets were synthesized by ultrasonication. Briefly, 100 mg of bulk C_3_N_4_ was added to 20 mL of water in a 50 mL centrifuge tube. The mixture was sonicated for 2 h using probe sonication at the power of 300 W. The ultrasound probe worked for 4 s in intervals of 2 s. The temperature of the sample solution was kept below 20 °C by an ice water bath. The resulting dispersion was centrifuged for 10 min at 1000 rpm to remove multilayered C_3_N_4_ nanosheets. Then, the supernatant was centrifuged at 12,000 rpm for another 10 min. The obtained precipitate, i.e., C_3_N_4_ nanosheets, was then re-dispersed in water for further use.

### 3.3. Characterization

The formation and purity of C_3_N_4_ nanosheets were examined by an X-ray diffractometer (XRD, Rigaku, Tokyo, Japan) in a 2θ range of 10–50°. The surface morphology was characterized by a transmission electron microscope (TEM, FEI Tecnai G2 F20, Hillsboro, OR, USA). The morphology of bacteria was characterized by a scanning electron microscope (SEM, TESCAN MIR4, Brno, Czech Republic).

### 3.4. Treatment of Wastewater by C_3_N_4_ and Ofx

To evaluate the effect of C_3_N_4_ on bacterial abundance and antibiotic-relative gene levels, fresh wastewater from hospital effluents was sampled from the Tianjin Hospital, with total organic matter at 150 mg/L. The wastewater was added into a 250 mL volume conical flasks to a liquid volume of 100 mL per flask. The flasks were randomly divided by four groups (n = 3), i.e., the control group in which the wastewater was used without the addition of both C_3_N_4_ and Ofx, the C_3_N_4_ group in which C_3_N_4_ (final concentration 80 ppm) alone was added into the wastewater, the C_3_N_4_+Ofx group in which C_3_N_4_ (80 ppm) and Ofx (16 ppm) were added, and the Ofx group in which Ofx (16 ppm) alone was added. The flasks were then shaken at 30 °C and 140 rpm in an incubator for the indicated time, and then the wastewater was sampled for assays of bacterial abundance and antibiotic-resistant gene levels.

#### 3.4.1. Assays of Bacterial Abundance

To measure the abundance of total living bacteria and Ofx-resistant bacteria, the wastewater samples from the flasks were diluted by a PBS buffer with a ten-fold gradient. The diluted samples were then added onto LB agar plates (trypsin 1%, NaCl 1%, yeast extract 0.5%, agar 2%) to culture the total living bacteria, and onto LB agar plates containing 16 ppm of Ofx to culture Ofx-resistant bacteria. After incubation at 30 °C for 24~48 h, the colony forming units on the agar plates were counted.

#### 3.4.2. Assays of Antibiotic-Resistant Genes (ARGs)

To evaluate the levels of representative ARGs, including *qnrS*, *ampC*, *mexB*, *mexD*, and *blaTEM*, total DNAs of the wastewater samples were extracted by using bacterial DNA extraction kits (Solarbio, Beijing, China). The levels of the ARGs in the obtained DNA samples were detected by using corresponding real-time PCR primers (Table S1), together with the SYBR Green Master Mix (QIAGEN, Hilden, Germany). The copies of ARGs were quantified by Rodriguez-Mozaz’s method. Then, 16S rDNA in each sample was also detected using the primers listed in Table S1 as the reference control.

### 3.5. In Vitro Antibacterial Assays

The *S. aureus* strains SA and ORSA were cultured in liquid LB medium overnight. The cells were then suspended with LB medium (OD_600_ = 0.01) in a 96-well plate with a series concentration of C_3_N_4_, Ofx and C_3_N_4_+Ofx. For C_3_N_4_, the concentrations were 10, 20, 40, 80 and 160 ppm, respectively. For Ofx, the concentrations were 0.5, 1, 2, 4, 8, 16 and 32 ppm, respectively. For C_3_N_4_+Ofx, the concentration of C_3_N_4_ was 80 ppm, and the concentrations for Ofx were 0.5, 1, 2, 4, 8, 16 and 32 ppm, respectively). The cells were cultured at 37 °C for 12 h. The absorbance at 450 nm was measured using a fluorescence microplate reader (PerkinElmer LLC Enspire, Waltham, MA, USA).

For SEM observation, the bacterial cells were fixed by 4% formaldehyde for 2 h, gradient-dehydrated by ethanol (30, 50, 70, 90 and 100% of volume fraction, respectively), freeze-dried, and observed by SEM [40].

### 3.6. Cell Viability Assay

The RAW 264.7 macrophages were cultured in DMEM medium, including 10% (*v/v*) fetal bovine serum (Gibco, Waltham, MA, USA), penicillin (100 U/mL), and streptomycin (100 U/mL), in a humidified atmosphere of 5% CO_2_ at 37 °C. The cells were seeded in a 96-well plate and incubated 24 h. Then, different amounts of C_3_N_4_ and Ofx were added into the cell cultures, and the cells were incubated for another 24 h. Cell viability was detected by the CCK-8 assay kits (Solarbio, Beijing, China).

### 3.7. Bacterium-Macrophage Interaction

The effect of C_3_N_4_, Ofx and C_3_N_4_+Ofx on the SA–macrophage interaction was evaluated by propidium iodide (PI)/Hoechst 33342 staining. Briefly, 1 × 10^5^ RAW 264.7 macrophages were infected by 5 × 10^5^ SA cells, followed by the addition of the agents (80 ppm C_3_N_4_, 16 ppm Ofx, and both). The mixture was cultured at 37 °C for 4 h and further stained by Hoechst 33342 and PI. Then, the cells were observed with a fluorescence microscope (OLYMPUS BX53F, Tokyo, Japan).

### 3.8. Adsorption of Ofx and Chloramphenicol

To quantify the capability of C_3_N_4_ nanosheets to adsorb the antibiotics (i.e., Ofx and chloramphenicol), the UV absorption of the two antibiotics was measured [41]. The absorption at 289 nm (Ofx) and 278 nm (chloramphenicol) increased with the antibiotic concentration, which could be used to make a calibration curve. After the incubation of C_3_N_4_ and Ofx (or chloramphenicol) for 12 h, the amounts of unbound antibiotics remained in the supernatant, which could be quantified using its absorbance at 289 nm (or 278 nm) and the calibration formula. Then, the amounts of adsorbed antibiotics were calculated.

### 3.9. Fluorescence Detection of Rhodamine 6G (Rho) Uptake and Efflux

Fluorescence detection of the uptake and efflux of Rho was assessed using a reported method with a slight modification [42]. SA (10^8^ cells) in LB with different treatments (with or without 80 ppm C_3_N_4_) was suspended in 2 mL PBS and incubated at 37 °C with constant shaking for 2 h. Then, Rho (10 μM) was added and incubated for 1 h. After centrifugation, the uptake of Rho was evaluated by testing the fluorescence intensity of the supernatant (excitation wavelength 530 nm, emission wavelength 556 nm) by using a fluorescence microplate reader. To test the efflux of Rho, glucose (4 mM) was added and incubated for another 1 h with constant shaking at 37 °C. After centrifugation, the fluorescence intensity of the supernatant was tested to quantify the efflux amount of Rho.

### 3.10. Investigation of Ofx Tolerance Mechanism

To investigate the possible Ofx tolerance mechanism in the presence of C_3_N_4_, SA (10^8^ cells) in LB with different treatments, including the control group, 80 ppm C_3_N_4_, 16 ppm Ofx, and C_3_N_4_+Ofx (80 ppm C_3_N_4_ and 16 ppm Ofx), was studied by several tests described below. Moreover, the number of SA strains in the suspensions was tested by colony forming unit (CFU) assays in LB solid plates.

#### 3.10.1. Membrane Potential Tests

Membrane potential changes in the bacteria with different treatments were detected using a BacLight Bacterial Membrane Potential Kit (Invitrogen, Waltham, MA, USA). Initially, SA (OD_600_ = 0.5) was incubated with C_3_N_4_, Ofx, and C_3_N_4_+Ofxfor 2 h. The bacteria were then collected, washed and re-dispersed in PBS. Bis-(1, 3-dibutylbarbituric acid) trimethine oxonol (DiBAC_4_(3)) was added to all samples at a final concentration of 8 μM and stained for 15 min. The fluorescence intensity of the samples was assessed using a microplate reader (excitation wavelength 488 nm, emission wavelength 520 nm).

#### 3.10.2. ATP Test

ATP levels in the bacteria with different treatments were detected using an ATP Assay Kit (Beyotime, Nantong, China). SA (OD_600_ = 0.5) in liquid LB medium was incubated with C_3_N_4_, Ofx, C_3_N_4_+Ofx and PBS (the control group) for 1 h. The bacteria were collected and lysed using a lysis buffer. After centrifugation, 10 μL of the supernatant was added to 100 μL of the test solution and incubated for 5 min. The luminescence was recorded using a fluorescence microplate reader. A standard curve was generated by measuring the luminescence of the ATP solution (10, 20, 40, 80, 100, 150, 200 nM).

#### 3.10.3. Rifampicin (Rif) Persister Assays

An amount of 100 μL of SA (OD_600_ = 0.5) was incubated with Rif (0.1 ppm) at 37 °C in a 96-well microplate for 15 min, and then C_3_N_4_, Ofx, C_3_N_4_+Ofx and PBS (the control group) were added to each well for 30 min. Then, the mixture was diluted 10,000 times and the number of SA strains in the suspensions was determined by CFU assays in LB solid plates.

#### 3.10.4. Arsenate (Ars) and Rif Persister Assays

An amount of 100 μL of SA (OD_600_ = 0.5) was incubated with Rif (0.1 ppm) at 37 °C in a 96-well microplate for 15 min, then Ars (1 mM) was added to each well, and C_3_N_4_, Ofx, C_3_N_4_+Ofx and PBS (Control group) was added to each well for 30 min. Then, the mixture was diluted 10,000 times, and the number of SA strains in the suspensions was determined by CFU assays in LB solid plates.

#### 3.10.5. Menadione (MD)-Ofx Survival Assays

An amount of 100 μL of SA (OD_600_ = 0.5) was incubated with MD (10 ppm) at 37 °C in a 96-well microplate for 15 min, and then Ofx and PBS (the control group) were added to each well = for 30 min. Then, the mixture was diluted 10,000 times and the number of SA strains in the suspensions was determined by CFU assays in LB solid plates.

#### 3.10.6. ROS Production Test

An amount of 100 μL of SA (OD_600_ = 0.5) was incubated with C_3_N_4_ and Ofx at 37 °C in a 96-well microplate for 2 h, with no treatment as the control. Then, 1 μM 2′,7′-dichlorofluorescin diacetate (DCFH-DA) was added and the cells were further incubated for 0.5 h. The fluorescence intensity of 520 nm was measured by a microplate reader (ex = 488 nm).

#### 3.10.7. Sodium Ascorbate (AAS)-Ofx Survival Assays

An amount of 100 μL of SA (OD_600_ = 0.5) was incubated with AAS (1 mM) at 37 °C in a 96-well microplate for 15 min, and then C_3_N_4_, Ofx, C_3_N_4_+Ofx and PBS (Control group) were added to each well for 30 min. Then, the mixture was diluted 10,000 times and the number of SA strains in the suspensions was determined by CFU assays in LB solid plates.

### 3.11. Statistical Analysis

Each experiment was performed with three replicates, and the values are shown with means ± SD. Differences between groups were compared by a one-way analysis of variance (ANOVA) (*p* < 0.05). All statistical tests were performed using the SPSS software package (Version 20, IBM, Armonk, NY, USA).

## 4. Conclusions

In summary, this study demonstrates an unintended consequence of bacterial exposure to C_3_N_4_ nanosheets, an ideal candidate with which to construct a photocatalyst to be widely used. The presence of C_3_N_4_ made *Staphylococcus aureus* more tolerant to ofloxacin, mainly through the mechanisms of ATP depletion and ROS generation. Consequently, incorrect discharge of C_3_N_4_ into the environment or manmade systems may have an impact that should not be underestimated. Future C_3_N_4_-based applications also need to consider their impact on the environment.

## Data Availability

Data may be available from the corresponding author, L.S., upon request.

## Supplementary Materials

The following supporting information can be downloaded at https://www.mdpi.com/article/10.3390/molecules29235780/s1: Figure S1: Viability of the SA and ORSA strains in the presence of C3N4; Figure S2: Viability of RAW 264; Figure S3: Chloramphenicol adsorption by C3N4; Figure S4: CFU results in the presence and absence of Rif with different treatments; Table S1: Real-time PCR primers used in this study.
