# Supplementary material for: Graphitic Carbon Nitride Confers Bacterial Tolerance to Antibiotics in Wastewater Relating to ATP Depletion
Source: Molecules. 2024 Dec 6;29(23):5780. doi: 10.3390/molecules29235780 (PMC11643725; doi:10.3390/molecules29235780)
Supplement: Supplementary file 1 [file molecules-29-05780-s001.zip › molecules-3332114-supplementary.pdf]

Graphitic Carbon Nitride Confers Bacterial Tolerance to Antibiotics in Wastewater Relating to ATP Depletion

Shuo Liu [1,*], Lin Teng [1] and Jiantao Ping [2,*]

[1] School of Energy and Chemical Engineering, Tianjin Renai College, Tianjin 301636, China
[2] Shandong Analysis and Test Center, Qilu University of Technology (Shandong Academy of Sciences), Jinan 250014, China
* Correspondence: liushuo@nankai.edu.cn (S.L.); pingjt@qlu.edu.cn (J.P.)

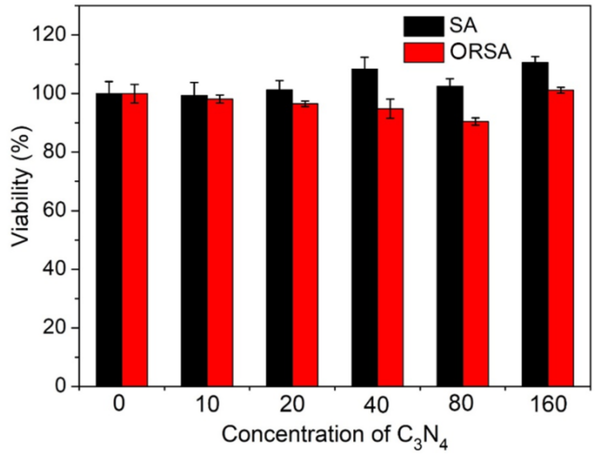

Figure S1. Viability of the SA and ORSA strains in the presence of C_3N_4.

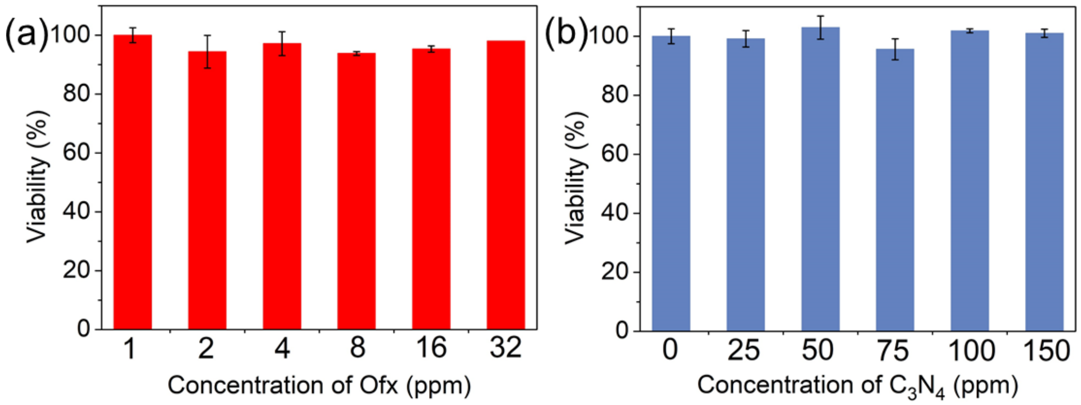

Figure S2. Viability of RAW 264.7 macrophages in the presence of (a) Ofx and (b) C_3N_4.

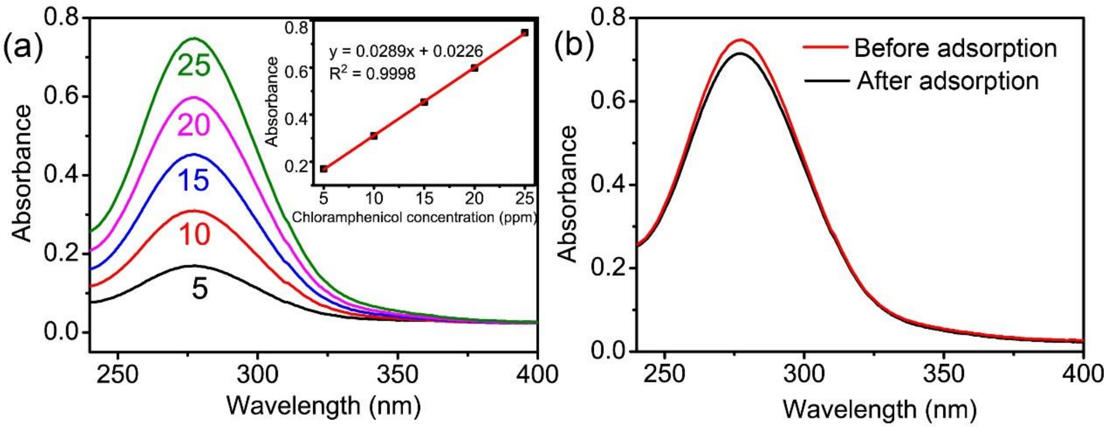

Figure S3. Chloramphenicol adsorption by C_3N_4. (a) UV-Vis spectra of chloramphenicol solutions with increased concentrations (Insert: Calibration curve obtained from the absorbance at 278 nm against the corresponding concentration of chloramphenicol, and the curve was fitted with a linear function.). (b) UV-Vis spectra of chloramphenicol solutions before and after C_3N_4 adsorption.

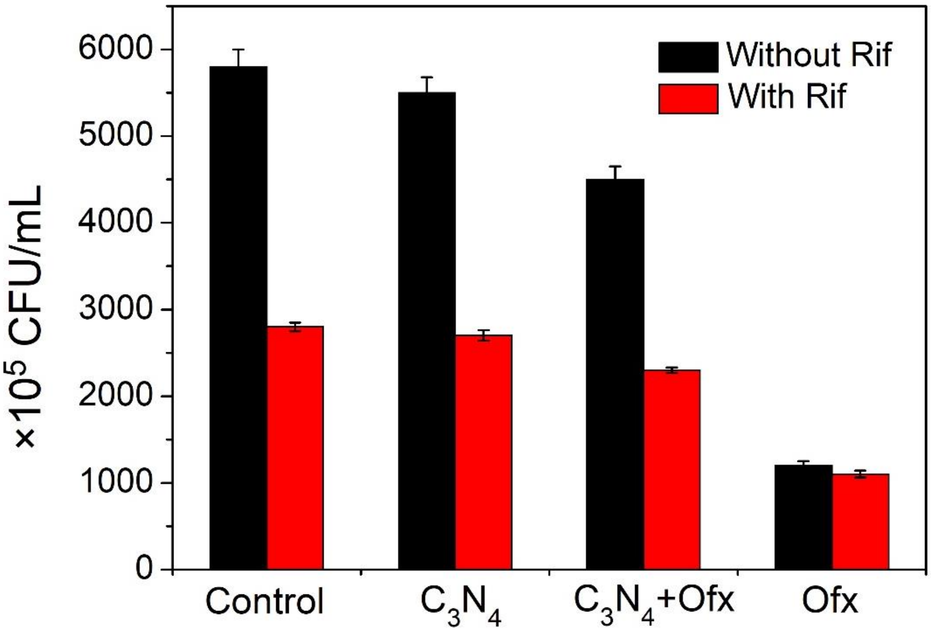

Figure S4. The CFU results in the presence and absence of Rif with different treatments.

Table S1. Real-time PCR primers used in this study

Gene	Primer name	Sequence (5'-3')
ampC	ampC-F	GGGCTGGCCTCGAAAGAGGAC
	ampC-R	GCACCGAGTCGGGGAACTGCA
mexB	mexB-F	CAAGGGCGTCGGTGACTTCCAG
	MexB-R	ACCTGGGAACCGTCGGGATTGA
mexD	mexD-F	GGAGTTCGGCCAGGTAGTGCTG
	MexD-R	ACTGCATGTCCTCGGGGAAGAA
bla_{TEM}	bla_{TEM}-F	CATTTCCGTGTCGCCCTTATTC
	bla_{TEM}-R	CGTTCATCCATAGTTGCCTGAC
qnrS	qnrS-F	GACGTGCTAACTTGCGTGAT
	qnrS-R	TGGCATTGTTGGAAACTTG
16S rDNA	F1048	GTGSTGCAYGGYTGTCGTCA
	R1194	ACGTCRTCCMCACCTTCCTC
